# Evaluation of a urogenital schistosomiasis behavioural intervention among students from rural schools in Unguja and Pemba islands, Zanzibar

**DOI:** 10.1016/j.actatropica.2021.105960

**Published:** 2021-08

**Authors:** Bobbie Person, David Rollinson, Said M. Ali, Ulfat A. Mohammed, Faiza M. A'kadir, Fatma Kabole, Stefanie Knopp

**Affiliations:** a Consultant of the Schistosomiasis Consortium for Operational Research and Evaluation, University of Georgia, Atlanta, Georgia, USA; b Wolfson Wellcome Biomedical Laboratories, Department of Life Sciences, Natural History Museum, Cromwell Road, London SW7 5BD, United Kingdom; c Public Health Laboratory - Ivo de Carneri, P. O. Box 122, Chake Chake, Pemba, United Republic of Tanzania; d Neglected Diseases Program, Ministry of Health, P.O. Box 236, Zanzibar Town, Unguja, United Republic of Tanzania; e Swiss Tropical and Public Health Institute, Socinstrasse 57, 4051 Basel, Switzerland; f University of Basel, Petersplatz 1, 4003 Basel, Switzerland

**Keywords:** Behaviour change, Elimination, Intervention, Schistosomiasis, *Schistosoma haematobium*, Zanzibar, EKBB, Ethikkommission beider Basel, HBM, Health Belief Model, HEBC, health education and behaviour change, MDA, mass drug administration, NHEBC, students not exposed to the HEBC interventions, SCORE, Schistosomiasis Consortium for Operational Research and Evaluation, ZAMREC, Zanzibar Medical Research Ethics Committee, ZEST, Zanzibar Elimination of Schistosomiasis Transmission

## Abstract

Urogenital schistosomiasis is a common experience among children in Zanzibar. There is a paucity of behavioural science-based, health education and behaviour change (HEBC) interventions for school-aged children, those at greatest risk for urogenital schistosomiasis. We assessed the influence of a HEBC intervention, guided by the Health Belief model, among rural schoolchildren on Pemba and Unguja islands in Zanzibar, Tanzania. From 2012 to 2016, a cluster-randomized trial to assess three different interventions against urogenital schistosomiasis was conducted in 90 schools and shehias across Zanzibar. The HEBC intervention was implemented in 15 schools per island. In 2017, at the trial conclusion, we administered written questionnaires to schoolchildren from 4 HEBC intervention schools and 4 not HEBC exposed schools on each island, respectively. Responses were compared between students that were exposed or not exposed to the HEBC intervention using a Fisher's exact test. A total of 1451 students, 708 from intervention and 743 from non-intervention schools completed the questionnaire. Noting some between island differences, students who had received the HEBC interventions reported significant improvements in knowledge about *Schistosoma haematobium* transmission and personal risk, strategies for schistosomiasis prevention, and self-reported changes in risk behaviours: stopped washing laundry/dishes 49.4% (350/708) *versus* 5.8% (43/743), stopped bathing in streams/ponds 49.4% (350/708) *versus* 4.2% (31/743), and stopped playing in streams/ponds 40.8% (289/708) *versus* 10.8% (80/743). HEBC exposed children also reported a significant increase in swallowing tablets during mass drug administration (MDA) campaigns (*when they had not before)* 30.2% (214/708) *versus* 4.6% (34/743). The school based HEBC interventions were associated with desirable positive behaviour change among students. Data suggest that scaling up HEBC interventions to all schools in high-risk areas, augmented with bi-annual MDA, can help to reduce prevalence of urogenital schistosomiasis in Zanzibar, strengthening the possibility for future disease elimination.

## Highlights

• Children self-reported changes in urogenital schistosomiasis risk taking behaviours.
• Children self-reported an increase in swallowing anthelmintic drugs during MDA.
• Rebranding the concept of “worm” to “blood fluke” created a critical perceived health threat.
• The Health Belief Model was a viable foundation for the behavioural intervention.

## 1 Introduction

More than 200 million people globally are estimated to be infected by blood flukes of the genus *Schistosoma* (Steinmann et al., 2006) and the global burden due to the disease schistosomiasis was estimated at 1.43 million disability-adjusted life years in 2017 (GBD and HALE collaborators, 2018). School-aged children living in endemic areas are particularly vulnerable to infection and disease due their frequent contact with natural open freshwater containing intermediate host snails that contribute to parasite transmission. The urogenital form of schistosomiasis is caused by the blood fluke species *S. haematobium*. The adult female parasite deposits its eggs in the veins around the bladder from where they can be easily transported to the genital and reproductive tract, potentially causing lesions that can result in female infertility or facilitate transmission of sexual infections (Gryseels, 2012). Urogenital schistosomiasis, if left untreated, can lead to severe health consequences such as female and male genital schistosomiasis, infertility, renal failure, and bladder cancer (Kjetland et al., 2012; Colley et al., 2014; Bustinduy et al., 2017).

The Schistosomiasis Consortium for Operational Research and Evaluation (SCORE) supported a clustered randomized trial in Zanzibar, which aimed to assess the effectiveness of three urogenital schistosomiasis control interventions for reducing and eliminating disease and infection (Knopp et al., 2012; Knopp et al., 2019a; Knopp et al., 2019b).The three interventions arms included: biannual mass drug administration (MDA) with praziquantel (Arm 1); biannual MDA and snail control (Arm 2); and biannual MDA and health education and behaviour change (HEBC) activities (Arm 3). The trial was conducted in 45 administrative areas (shehias) on Unguja and Pemba islands, respectively, from 2011 until 2017 (Knopp et al., 2012; Knopp et al., 2013; Knopp et al., 2019a; Knopp et al., 2019b).

The HEBC interventions for Arm 3 of the trial were designed using a Human Centred Design approach whereby community members, including teachers and students, assisted in the design of the interventions (Celone et al., 2016; Person et al., 2016a; Person et al., 2016b). Formative research findings obtained in Zanzibar in 2011 were shared with community members and helped to inform the intervention co-design process (Person et al., 2016b). This participatory process resulted in three community co-designed intervention components deemed as potentially useful for preventing *S. haematobium* transmission among students and community members (Celone et al., 2016; Person et al., 2016b): 1) school-wide, classroom-based HEBC interventions focusing on new interactive teaching methods and classroom-based tools, materials, and activities for teachers to educate students about *S. haematobium* transmission, treatment, prevention, and behaviour change options; 2) school-wide, outdoor-based interventions focusing on safe play with HEBC about *S. haematobium* transmission, treatment, prevention, and behaviour change options; 3) community-based structural interventions dedicated to the provision of urinals to avoid contamination of natural open freshwater bodies with parasite eggs contained in urine and laundry washing platforms to prevent infection of humans with *S. haematobium* cercariae.

The intervention components were further refined using theoretical constructs from the Health Belief Model (HBM) to guide the development of intervention components, including perceived threat of disease, perceived benefits and barriers to disease prevention, self-efficacy, and cues to action that are meant to increase awareness and readiness to change along with a Social Ecological framework to guide implementation activities (Glanz and Rimer, 1995; Champion and Skinner, 2008; Painter et al., 2008; Glanz and Bishop, 2010; Carico et al., 2021). In the evaluation presented here, we aimed to assess the impact of the school-based HEBC interventions on schistosomiasis knowledge; perceived threat and perceived susceptibility to disease; self-efficacy to prevent and seek disease treatment; risky behaviors; uptake of anthelminthic tablets provided during mass drug administration (MDA); and positive protective behaviors of schoolchildren exposed to the interventions in Arm 3 of the trial compared with children from Arm 1 or Arm 2 who did not receive the HEBC.

## 2 Methods

### 2.1 Ethical considerations

This research was conducted as part of the behaviour change activities of the cluster randomized trial approved by multiple institutional and ethical review boards: the Zanzibar Medical Research Ethics Committee in Zanzibar, United Republic of Tanzania (reference no. ZAMREC 0003/Sept/011), the “Ethikkommission beider Basel” (EKBB) in Switzerland (reference no. 236/11), and the Institutional Review Board of the University of Georgia in Athens, Georgia, United States of America (project no. 2012-10138-0). Written informed consents were obtained from the parents of all students participating in the behavioural evaluation.

### 2.2 Study area and population

Unguja and Pemba islands are part of the Zanzibar archipelago off the coast of the United Republic of Tanzania. The projected resident population of Zanzibar for 2020 was 1.6 million (OCGS, 2020). Islam is the predominant religion. Unguja and Pemba islands are separated by a 50-kilometer-wide ocean channel. Pemba, smaller than Unguja, had a land mass of land area of 988 square kilometres with a population of 406,848 people in 2012, when the last population census was conducted (OCGS, 2013). Most of this rural island, which is hillier and more fertile than Unguja, is dominated by small-scale farming. There is also large-scale farming of cash crops such as cloves. Pemba has far less tourism than Unguja in part due to the conservative Islamic nature of the population and few hotels and lodges.

Unguja has an overall area of about 1,666 square kilometres with a population size of 896,721 inhabitants in 2012 (OCGS, 2013). The population is typically concentrated in the urban region. Unguja has a robust tourism industry. While Kiswahili is the predominant language, there are Kiswahili dialects specific to each island. Both Unguja and Pemba islands are divided into districts: six districts (at the time of the study) on Unguja and four districts on Pemba. *S. haematobium* transmission occurs in all districts of Unguja except the South district and in all four districts of Pemba (Stothard and Rollinson, 1997; Stothard et al., 2000; Stothard et al., 2002). The districts are sub-divided into small administrative units called shehias. Most shehias have at least one primary school. Our study included students from 16 among the 90 government primary schools that were part of the cluster randomized trial.

### 2.3 Research team

The behavioural research team that conducted the HEBC interventions and assessment reported here consisted of a senior social scientist and three local Kiswahili and English-speaking research assistants from the Neglected Diseases Program of the Ministry of Health on Unguja, and three research assistants from the Public Health Laboratory-Ivo de Carneri on Pemba. The research assistants were trained in research ethics and data collection methods by the senior social scientist and served as the logistic coordinators and primary data collectors working with the school headmaster, classroom teachers, parents, and students for this evaluation.

### 2.4 Study design

From 2012 to 2017, a cluster-randomized trial to assess three different interventions against urogenital schistosomiasis (locally known as *kichocho*) was conducted in 90 schools and shehias across Zanzibar (Knopp et al., 2012; Knopp et al., 2013). Among them, the HEBC intervention was implemented in 30 schools, i.e. 15 schools per island and is described elsewhere in detail (Person et al., 2016b). For the evaluation of the impact of the HEBC interventions, we selected 8 schools per island. Four schools on each island belonged to the HEBC study Arm 3, and two schools to Arm 1 and Arm 2, respectively, which had not received the HEBC. For inclusion, in a first step, 6 primary schools on each island, with the highest *S. haematobium* prevalence in 2016, were identified from the 15 study schools that received the HEBC in Arm 1. Subsequently, in a second step, 4 among the 6 schools were randomly selected. Additionally, two primary schools in Arm 1 and two schools in Arm 2 with the highest *S. haematobium* prevalence that had not received the HEBC intervention were selected to serve as comparison group. We choose the highest prevalence schools because we thought they would be the most challenging for changing behaviour.

All children in grade 4 and grade 6 classrooms in the selected schools on Pemba and Unguja, respectively, were enrolled. Written informed consents, approved by all school headmasters, were obtained from the parents of all students in all participating evaluation classrooms. Only students who were in attendance the day of the questionnaire with signed informed consents were included in the evaluation. Responses were compared between students that were exposed (HEBC) or not exposed (NHEBC) to the interventions.

### 2.5 Data collection

Informed consent documents, a brief questionnaire with in-depth instructions on how to administer the instrument, along with the sampling frame were developed in English by the behavioural research teams on each island in collaboration with the senior social scientist. The informed consent documents and brief questionnaire with several open-ended questions were translated into Kiswahili. The anonymous questionnaire included demographics, previous episodes of schistosomiasis, missing school-based MDA, exposure to classroom and outdoor HEBC, understanding of risk behaviours, illness in the body, treatment options, self-described disease risks for children, exposure to contaminated water sources, identification of the intervention component that was most influential for creating personal change, and self-reported behaviours changed since being part of the intervention. Some of the questions were in the form of pictures rather than words due to lower literacy levels in grade 4. The questionnaire had two open-ended questions. The questionnaire was pilot-tested and revised before finalizing it for data collection.

The behavioural research teams, under the supervision of the senior social scientist, were responsible for implementing all evaluation activities. Each team was trained on how to administer the questionnaire and had a guidance document on how to present each question to the students. One team member went to each classroom and with the help of the teacher collected the parental signed consent forms, and subsequently distributed the pencil-paper questionnaires to all students. A member of the team stood in the classroom and slowly read each question out loud and students were asked to independently tick the correct answer or write in their answer for open-ended questions. Students were allowed to skip questions they did not want to answer. Students were instructed to hand in the questionnaire to a team member or teacher upon their completion of the questionnaire. It took approximately 20 minutes for most students to complete the questionnaire. Unexpectedly, a few students from grade 4 classrooms had difficulties reading and writing Kiswahili. A team member assisted these students by privately reading the questions out loud and scribed their answers separate from the classroom. Data were collected over a 4-week period in February 2017 while school was in session and under the supervision of the senior social scientist.

### 2.6 Data management and analysis

The Kiswahili questionnaires were entered into an Excel spreadsheet (Microsoft Corporation, 2016) and then into an English mask in Epi-Info version 7 (Centers for Disease Control and Prevention, Atlanta, United States of America) where data cleaning, exploration, and frequencies were run. Using cleaned data, a univariate analysis was conducted using Fisher's exact test with p-values of <0.05 considered statistically significant given the smaller number of respondents.

## 3 Results

### 3.1 Characteristics of the student study populations on Pemba and Unguja

Data findings were derived from analyses of a total of 1451 student questionnaires from 8 schools on Pemba and 8 schools on Unguja, respectively. A total of 748 students on Pemba and of 703 students on Unguja, respectively, completed the brief questionnaire. On Pemba there were 389 HEBC students (228 girls and 161 boys) and 359 NHEBC students (204 girls and 155 boys) enrolled in the study. On Unguja there were 319 HEBC students (195 girls and 124 boys) and 384 NHEBC students (222 girls and 162 boys) enrolled in the study. There were no significant differences between children in intervention and comparison schools on either island by number of years in school, or median age in each respective grade.

The median age of grade 4 and 6 girls combined for Pemba was 13 years (range: 9-16) and Unguja was 12 years (range: 9-16). The median age for Pemba grade 4 and 6 boys combined was 13 years (range: 9-16) and Unguja was 12 years (range: 9-16).

### 3.2 Students’ access to tap water, previous infection with S. haematobium and MDA participation

Among all participants from both islands, 68.0% (483/708) of all HEBC and 72.1% (536/743) of NHEBC students reported unreliable access to clean tap water. On Pemba, 22.4% (87/389) of HEBC and 18.1% (65/359) of NHEBC students reported having had at least one previous *S. haematobium* infection since grade 1. On Unguja, 20.4% (65/319) of HEBC and 34.9% (134/384) of NHEBC students reported at least one previous *S. haematobium* infection since grade 1.

Most students received anthelmintic drugs provided during school-based MDA campaigns. However, there were students on both islands who reported being absent during school-based MDA. On Pemba 15.7% (61/389) of HEBC and 14.2% (51/359) NHEBC students reported being absent at least once during an MDA drug distribution. On Unguja, 8.8% (28/319) of HEBC and 19.5% (75/384) of NHEBC students reported being absent at least once during school-based MDA. Among students who were absent from school during the last school-based MDA, some students received their MDA drugs through community drug distributors while being at home. Others did not take any drugs during the MDA campaign when absent from school. Among students who were absent from school during the last school-based MDA, 57.4% (35/61) of HEBC and 64.7% (33/51) of NHEBC students from Pemba failed to swallow any MDA tablets. Similarly, on Unguja, 64.3% (18/28) of HEBC and 50.7% (38/75) of NHEBC students that did miss MDA at school failed to swallow any MDA tablets.

### 3.3 Students’ exposure to natural open freshwater

Students were asked to identify household chores or activities that parents asked them to perform that could expose them to natural open freshwater, potentially contaminated with *S. haematobium* cercariae. HEBC and NHEBC students across both islands identified watching siblings and bathing them in the river, working in the field and using local fresh water sources to water the crops, fetching water from the river, tending animals which included taking them to local water sources, and washing laundry in the river as the top activities that would put them at risk for getting kichocho.

### 3.4 Students‘ schistosomiasis knowledge, risk perceptions, and threats to health

All students were given a set of multiple choice questions related to kichocho knowledge on disease transmission, identification of what lives inside your body that makes you sick with kichocho, and what treatment cures kichocho. Findings from Pemba HEBC students compared to NHEBC students are reported in Table 1 and from Unguja HEBC compared with NHEBC students are shown in Table 2. The p value is significant at 0.05 suggesting that there is a 95% probability that differences between HEBC and NHEBC are due to exposure to the intervention rather than a random chance occurrence. Female and male students from HEBC schools on both islands were significantly more likely to report knowledge that the blood fluke parasite was the threat to their health rather than the snail in the river. Interestingly, Pemba NHEBC boys displayed a high level of awareness with 66.8% (102/155) reporting that the blood fluke caused illness in their body. While the percent of student responses was high in both groups, reporting that washing clothes in the river was perceived as a risk for kichocho infection was significantly higher in the HEBC students on both islands. All students typically reported that praziquantel was the best medicine to treat kichocho.

**Table 1:** Pemba students’ schistosomiasis knowledge, risk perceptions, and threats to health

	Pemba HEBC girls (n=228)		Pemba NHEBC girls (n=204)			Pemba HEBC boys (n=161)		Pemba NHEBC boys (n=155)		
	Freq.	Percent	Freq.	Percent	p-value	Freq.	Percent	Freq.	Percent	p-value
Blood fluke causes illness	223	97.8%	41	20.1%	p<0.001	149	92.6%	102	66.8%	p<0.001
Snail causes illness	1	0.04%	147	72.1%	p<0.001	1	0.06%	41	26.5%	p<0.001
Washing clothes in river risk for disease	223	97.8%	176	86.3%	p<0.001	158	98.1%	136	87.7%	p<0.001
Praziquantel best medicine	225	98.7%	196	96.1%	p=0.079	161	100%	149.0	96.1%	p=0.013

**Table 2:** Unguja students’ knowledge and risk perceptions about disease transmission and threats to health

	Unguja HEBC girls (n=195)		Unguja NHEBC girls (n=222)			Unguja HEBCboys (n=124)		Unguja NHEBCboys (n=162)		
	Freq.	Percent	Freq.	Percent	p-value	Freq.	Percent	Freq.	Percent	p-value
Blood fluke causes illness	182	93.3%	75	33.8%	p<0.001	120	96.8%	45	27.8%	p<0.001
Snail causes illness	1	0.05%	130	58.6%	p<0.001	1	0.8%	111	68.5%	p<0.001
Washing clothes in river risk for disease	180	92.3%	191	86.0%	p<0.001	117	94.4%	143	88.3%	p<0.001
Praziquantel best medicine	189	96.9%	212	95.5%	p=0.310	119	96.0%	151	93.2%	P=0.230

We assigned a single variable to a combined set of correct answers: the blood fluke causes illness in the body, washing clothes in the river was a risk for disease, and praziquantel was the best medicine to treat schistosomiasis suggesting a more comprehensive understanding of the disease. On Pemba, 95.2% (217/228) of HEBC girls compared to 18.6% (38/204) of NHEBC girls and 90.7% (146/161) of HEBC boys compared to 59.4% (92/155) of NHEBC boys correctly identified the risk-knowledge variable of all three questions (p<0.001).

Similarly, on Unguja, 83.1% (162/195) of HEBC girls compared to 27.9% (62 /222) of NHEBC girls and 87.1% (108/124) of HEBC boys compared to 21.0% (34/162) of NHEBC boys correctly identified the risk-knowledge variable of all three questions (p<0.001).

There was a significant difference between HEBC and NHEBC among girls and boys between the islands. When comparing the HEBC intervention between the islands, 95.2% (217/228) of Pemba HEBC girls compared to 83.1% (162/195) of Unguja HEBC girls correctly identified the risk-knowledge variable of all three questions (p<0.001). Additionally, there was a significant difference between Pemba NHEBC girls at 18.6% (38/204) and Unguja NHEBC girls at 27.9% (62 /222) correctly identifying the risk-knowledge variable of all three questions (p<0.016). There was no significant difference between Pemba HEBC boys (90.7% (146/161)) compared to Unguja HEBC boys (87.1% (108/124)) correctly identifying the risk-knowledge variable (p=0.219). Although on Pemba, significantly more NHEBC boys (59.4% (92/155) compared with Unguja NHEBC boys (21.0% (34/162)) correctly identified the risk-knowledge variable of all three questions, suggesting a potential difference in intervention fidelity across intervention and comparison schools.

### 3.5 Students’ perceptions of activities that increase personal susceptibility to schistosomiasis transmission

Students were asked to list two risky behaviours where children like themselves might get infected with kichocho. On Pemba, shown in Table 3, washing items in a stream and bathing in a stream ranked as the top two risk activities for HEBC students while swimming followed by washing in the stream were the highest ranked activities for NHEBC students.

**Table 3:** Pemba students perceived risky behaviour for schistosomiasis transmission

Risk behaviours	Pemba HEBC girls (n=228)		Pemba NHEBC girls (n=204)		Pemba HEBC boys (n=161)		Pemba NHEBC boys (n=155)	
	Freq.	Percent	Freq.	Percent	Freq.	Percent	Freq.	Percent
Washing items in a stream	194	85.1%	17	8.3%	105	65.2%	27	17.4%
Swimming in a stream	171	75.0%	104	51.0%	105	65.2%	105	67.7%
Bathing in a stream	24	10.5%	31	15.2%	22	13.7%	9	5.8%

As shown in Table 4, Unguja HEBC girls and boys ranked bathing in the stream and then swimming highest, while NHEBC girls and boys ranked swimming and then bathing in the stream more frequently as risk activities for acquiring *S. haematobium* infection. “Dirty water or drinking dirty water” as a disease transmission source was listed by 36.9% (274/743) of all NHEBC as compared to 0.3% (2/708) of all HEBC students from both islands.

**Table 4:** Unguja students perceived risky behaviour for schistosomiasis transmission

Risk behaviours	Unguja HEBC girls (n=195)		Unguja NHEBC girls (n=222)		Unguja HEBCboys (n=124)		Unguja NHEBCBoys (n=162)	
	Freq.	Percent	Freq.	Percent	Freq.	Percent	Freq.	Percent
Bathing in a stream	107	54.9%	64	28.3%	51	41.1%	69	42.6%
Swimming in a stream	81	41.5%	116	52.3%	49	39.5%	84	51.9%
Washing items in a stream	56	28.7%	14	6.3%	32	25.8%	14	8.6%

### 3.6 Teachers talked about schistosomiasis in school

One requirement for fidelity to the HEBC intervention was teachers teaching and talking to their students about kichocho on a regular schedule. It was expected that teachers from non-intervention schools would also talk with their students because everyone received praziquantel tablets through bi-annual school-based MDA campaigns. Among the HEBC students from Pemba, 99.2% (386/389) reported that their teachers talked about kichocho and typically talked a median of 4 times in the past 6 months (range of 1-10). In contrast, among the NHEBC students from Pemba, 16.8% (60/358) reported their teacher talked about kichocho and typically talked a median of 2 times (range of 0-4).

Among HEBC students from Unguja, 96.9% (309/319) reported their teacher talked about kichocho and typically talked a median of 4 times in the past 6 months (range of 1-12). Among the NHEBC Unguja students, 79.4% (305/384) reported their teacher talked with a median of 2 times (range of 0-6).

### 3.7 Students’ self-reported reduction of risk behaviours and increase of positive protective behaviours

Table 5 is indicated here, Pemba HEBC girls and boys reported a significant increase of 49.6% and 40.4 % respectively, in now swallowing the anthelminthic tablets provided during MDA (*when they did not swallow the drugs in previous MDA efforts*). Also 21.9% of NHEBC boys reported an increase in swallowing the MDA tablets. HEBC girls reported significantly higher rates of change among stopped washing, bathing, swimming and playing in a stream than NHEBC girls. HEBC boys reported significantly higher rates of stopped bathing and washing in the stream than NHEBC boys, but there was no difference between groups having stopped swimming or playing in streams.

**Table 5:** Pemba students’ self-reported reduction of risk behaviours and increase of positive protective behaviours

	Pemba HEBC girls (n=228)		Pemba NHEBC girls (n=204)			Pemba boysHEBC (n=161)		Pemba NHEBC boys (n=155)		
Behaviours	Freq.	Percent	Freq.	Percent	p-values	Freq.	Percent	Freq.	Percent	p-values
Now swallow medicine	113	49.6%	0	0.0 %	p<0.001	65	40.4%	34.0	21.9%	p<0.001
Less time wash stream	30	13.2%	0	0.0%	p<0.001	38	23.6%	4	2.6%	p<0.001
Stop wash stream/pond	141	61.8%	0	0.0 %	p<0.001	58	36.0%	9	5.8 %	p<0.001
Less time bath stream	4	1.8%	0	0.0%	p=0.007	1	0.6%	2	1.3%	p=0.486
Stop bath stream/pond	117	51.3%	0	0.0 %	p<0.001	57	35.4%	20	12.9 %	p<0.001
Less swim/play stream/pond	5	2.2%	1	0.5%	p=0.136	3	1.9	15	9.7	P=.0020
Stop swim/play stream/pond	104	45.6%	0	0.0 %	p<0.001	74	46.0%	63	40.7%	p=0.200

As shown in Table 6, on Unguja, girls and boys in HEBC schools reported significantly higher rates of now taking praziquantel, stopped washing, bathing and playing/swimming in the stream than NHEBC students.

**Table 6:** Unguja students’ self-reported reduction of risk behaviours and increase of positive protective behaviours

	Unguja HEBC girls (n=195)		Unguja NHEBC girls (n=222)			Unguja HEBC boys (n=124)		Unguja NHEBC boys (n=162)		
Behaviours	Freq.	Percent	Freq.	Percent	p-value	Freq.	Percent	Freq.	Percent	p-value
Now swallow medicine	24	12.3%	0	0 %	p<0.001	12	9.7%	0	0 %	p<0.001
Less time bath stream	7	3.6%	0	0	P=0.005	6	4.8%	1	0.6%	P=0.028
Stop bath stream/pond	116	59.5%%	6	2.7%	p<0.001	60	48.4 %	5	3.1 %	p<0.001
Less wash time wash stream	30	15.4%	1	0.5%	p<0.001	22	17.7%	5	3.1%	p<0.001
Stop wash stream/pond	99	50.8 %	23	10.4%	p<0.001	52	41.9 %	11	6.8 %	p<0.001
Less time swim/play stream	7	3.6	3.	1.4%	P=0.121	2	1.6%	8	4.9%	P=0.115
Stop swim/play stream/pond	66	3.9 %	8	3.6 %	p<0.001	45	36.3 %	9	5.6 %	P<0.001

### 3.8 HEBC influential intervention tools and activities

All students that received the HEBC intervention were asked to list 3 best classroom-based or outdoor-based HEBC materials, exercises, and activities that were most influential in increasing their knowledge, perceptions of risk as well as their choice to reduce risky behaviours and adopt more positive protective behaviours.

As shown in Table 7, Pemba HEBC students listed the blood fluke picture and talk as the top most influential activity. HEBC girls further reported that the Kichocho Day events, snail board, and kichocho games were also in the top influential activities. HEBC boys reported that in addition to the blood fluke picture and talk, the snail board, the Bambo comic and Kichocho Day events were most influential activities. While HEBC intervention components were not supposed to be available to NHEBC students, it appeared that some students were exposed to several of the intervention educational materials.

**Table 7:** Pemba HEBC students’ most influential behavioural intervention activities

Intervention components	Pemba HEBC girls (n=228)		Pemba NHEBC girls (n=204)		Pemba boysHEBC (n=161)		Pemba NHEBC boys (n=155)	
	Freq.	Percent	Freq.	Percent	Freq.	Percent	Freq.	Percent
Blood fluke picture/talk	173	75.9%	0	0	128	79/5%	25	16.1%
Kichocho Day event	154	67.5%	0	0	41`	25.5%	0	0
Snail board activity	113	49.6%	0	0	82	50.9%	32	20.7%
Kichocho games	65	28.5%	0	0	25	15.5%	19	12.3%
Bambo comic	17	7.5%	21	10.3%	55	34.2%	13	8.4%
Teacher flipchart	22	9.7%	0	0	19	11.8%	0	0
Classroom teaching	4	1.8	0	0	5	3.1	0	0
Kichocho life cycle picture	32	14.0%	0	0	36	22.4%	1	0.7%%
Poems	7	3.1%	0	0	0	0	0	0
Video	22	9.7%	0	0	0	0	0	0

On Unguja, as shown in Table 8, kichocho games, teacher flipchart, classroom teaching, the blood fluke picture and talk and poems were the top ranked educational components reported by HEBC girls. Classroom teaching, teacher flipchart, kichocho games, and blood fluke picture and talk were tops among HEBC boys.

**Table 8:** Unguja HEBC students’ most influential intervention activities

Intervention components	Unguja HEBC girls (n=195)		Unguja NHEBC girls (n=222)		Unguja HEBC boys (n=124)		Unguja NHEBCboys (n=162)	
	Freq.	Percent	Freq.	Percent	Freq.	Percent	Freq.	Percent
Kichocho games	83	42.6%	0	0	52	41.9%	0	0
Teacher flipchart	90	46.2%	0	0	62	50.0%	1	0.6
Classroom teaching	75	38.5%	0	0	64	51.6%	2	1.2%
Blood fluke picture	47	24.1%	0	0	45	36.3%	0	0
Kichocho Day event	24	12.4%	0	0	19	15.3%	0	0
Poems	41	21.0%	0	0	16	12.9%	0	0
Bambo comic	14	7.2%	0	0	2	1.6%	0	0
Songs	8	4.1%	0	0	9	7.3%	0	0
Snail board activity	0	0	0	0	8	6.5%	0	0

On Pemba, 98.5% (383/389) of all HEBC students reported attending at least one Kichocho Day event with 54.5% (212 /389) reporting they attended 4 or more Kichocho Day events. On Unguja, 98.8% (315/319) of all HEBC students attended at least one Kichocho Day event with 63.6% (203/319) attending 4 or more events. HEBC students reported numerous safe play activities to prevent schistosomiasis including skipping rope 70.6% (500/708), Nage 52.8% (374/708), football 39.7% (281/708), Madako 31.8% (225/708), and tug-of-war 21.8% (154/708).

## 4 Discussion

School-aged children in Zanzibar regularly expose themselves to contaminated open natural freshwater bodies through household chores expected by their parents and their own recreational play. In order to reduce, control, and ultimately eliminate schistosomiasis, there is an increased recognition that HEBC interventions should be: 1) created in partnership with community members from the targeted population; 2) based on social and behavioural science to trigger behaviour change; 3) sustained over time; and 4) complimented by regular MDA with praziquantel (Asaolu and Ofoezie, 2003; Bruun and Aagaard-Hansen, 2008; Aagaard-Hansen et al., 2009; Glanz and Bishop, 2010). When piped, clean water is unavailable, such inclusive interventions along with regular MDA can provide risk mitigation to reduce prevalence of disease. Previous research has noted that top down approaches for designing and implementing HEBC and behaviour change interventions are often a poor fit for the population at risk for disease (Mwanga et al., 2004; Parker et al., 2008; Allotey et al., 2010; Parker and Allen, 2014). Lack of attention, especially to children's social, cultural, religious, environmental, and physical context, often results in inadequate intervention efforts that at best, only increase knowledge (Lewis et al., 1997). Increases in knowledge alone are rarely adequate to change a person's attitude and risk behaviours (Lewis et al., 1997).

The Human Centred Design approach, which guided the community-co-designed development of behaviour change interventions in Zanzibar, encouraged teacher, student, and parent participation in the design process and identification of the most efficacious implementation strategies for the school-based HEBC interventions. This approach created community member ownership and tailored the appropriateness of the interventions to local Islamic Zanzibari children (Person et al., 2016a).

Findings from our descriptive analysis found a significant association between exposure to our behavioural interventions against urogenital schistosomiasis, guided by the social ecological framework and grounded in constructs of the HBM and improvements in knowledge about *S. haematobium* transmission and perceptions of risk; improved attitudes towards prevention and treatment of the disease with an increased uptake of swallowing of anthelminthic tablets during MDA campaigns, suggesting that a group of children had been resistant to or did not swallow the drugs in previous MDA efforts prior to the intervention; and self-reported changes in behaviours.

The HBM previously used with other public health diseases to predict preventive health behaviours and to develop interventions, posits that several constructs in combination influence the possibility that a person would adopt a recommended positive change in health behaviour (Person et al., 2016a; Siddiqui et al., 2016; Tola et al., 2016; Patterson et al., 2018). In the HEBC intervention we wanted students to feel personally threatened by the disease, meaning they must feel personally susceptible to urogenital schistosomiasis and made aware of the serious or severe health consequences. Secondly, they must believe that the benefits of making change outweigh the perceived barriers to or costs of the change, and finally they must feel confident and capable of making the change with support of others (Glanz and Bishop, 2010). When these beliefs are held, cues to action such as songs, games, and drama can trigger behaviour change actions. Our previous qualitative research findings identified lack of knowledge about the blood fluke and the life cycle of the blood fluke associated with disease transmission and serious health consequences in the body as reasons for non-compliance with anthelminthic drug uptake and persistence of ongoing risky behaviours before the behavioural interventions were implemented (Person et al., 2016a).

Students exposed to the HEBC interventions reported that the most powerful motivators for changing behaviour and swallowing the MDA tablets were indeed these materials with images of the actual blood fluke, and the explanation drawing distinctions between the serious health outcomes associated with infection by blood flukes when compared to “normal” worms. In the past the *Schistosoma* blood fluke was characterized a worm. There was an existing social norm that everyone gets worms which were not perceived as a threat because there were few perceived significant health consequences of having worms (Celone et al., 2016; Person et al., 2016a; Person et al., 2016b). So, it was important to have children and adults be aware of the negative health outcomes associated with the urogenital schistosomiasis blood fluke, for the health outcomes to be severe, to perceive themselves to be susceptible to those negative outcomes, and to believe in the advantages of the recommended protective behaviours (Glanz and Rimer, 1995; Champion and Skinner, 2008; Painter et al., 2008). Therefore, we rebranded the *Schistosoma* “worm” into the “blood fluke” schistosome. We represented urogenital schistosomiasis with actual images of the blood fluke schistosome that students and adults found particularly scary. The image was accompanied with messages as to the serious health consequences caused by the blood fluke such as possible bladder cancer and reproductive problems when desiring children in the future. These images and messages were used for all educational tools, materials, games, and other teaching aids, which can be found on the Global Schistosomiasis Alliance website (https://www.eliminateschisto.org/resources/teacher-toolkit-for-urogenital-schistosomiasis). The schistosome was viewed as a very scary, ugly and disgusting creature and the perceived benefits of staying out of contaminated water and taking the medicine meant that you would not have these in your body. The prospect of a person taking action on a health recommendation often relies upon the perceived benefits minus perceived barrier to taking that action (Glanz and Rimer, 1995; Abraham and Sheeran, 2005; Champion and Skinner, 2008; Painter et al., 2008; Aagaard-Hansen et al., 2009; Orji et al., 2012). A group of Pemba boys in 3 among the 4 NHEBC schools reported being exposed to the HEBC blood fluke picture, the snail board, and safe play games for prevention of disease. Among them, 66.8% reported that the blood fluke lived in the body causing illness. When exploring these unexpected findings, we found out that an MDA health worker had taken materials from the HEBC intervention and used them in a stand-alone fashion with these boys.

Despite the increasing recognition for the need of science-based HEBC interventions to complement preventive chemotherapy, snail control, and improved water and sanitation for the prevention, control, and treatment of urogenital schistosomiasis, few exist (Utzinger et al., 2009; Rollinson et al., 2013). More science-based HEBC interventions need to be conducted and evaluated in order to provide an evidence-base for other countries that aim to reduce schistosomiasis as a public health problem.

The main limitation of this study was the potential biases associated with student self-reported HEBC activities, and changes in behaviors. Students may have exaggerated their responses or reported socially desirable answers. Identifying and/or validating the most critical construct for change went beyond the scope of this study. The HEBC intervention, while tailored to the Zanzibari schoolchildren, was predicated upon teachers trained by the HEBC team returning to their school and training all the other teachers in that school and may not be generalizable to all schools in Zanzibar. Despite the limitations stated above, we do believe that results were less likely to be influenced by those limitations.

## 5 Conclusion

The school-based intervention, developed using underlying theoretical constructs of the Health Belief model of health behaviour, was associated with an increase in swallowing all anthelminthic tablets provided during MDA campaigns. Additionally, students reported significant improvements in urogenital schistosomiasis knowledge, identification of personal risk behaviours, increased use of strategies for disease prevention, and self-reported changes in risk behaviours.

## Author statement

**Table:** 

Conceptualization	Bobbie Person, David Rollinson, Said M. Ali, Fatma Kabole, Stefanie Knopp
Methodology	Bobbie Person
Software	Not applicable
Validation	Not applicable
Formal analysis	Bobbie Person
Investigation	Bobbie Person, Ulfat A. Mohammed, Faiza M. A'kadir, Stefanie Knopp
Resources	David Rollinson, Said M. Ali, Fatma Kabole
Data Curation	Bobbie Person, Ulfat A. Mohammed, Faiza M. A'kadir
Writing - Original Draft	Bobbie Person
Writing - Review & Editing	Bobbie Person, David Rollinson, Said M. Ali, Ulfat A. Mohammed, Faiza M. A'kadir, Fatma Kabole, Stefanie Knopp
Visualization	Bobbie Person
Supervision	Bobbie Person, David Rollinson, Said M. Ali, Fatma Kabole, Stefanie Knopp
Project administration	Bobbie Person, David Rollinson, Said M. Ali, Fatma Kabole, Stefanie Knopp
Funding acquisition	David Rollinson, Stefanie Knopp

## Declaration of Competing Interest

Authors have declared that no competing interests exist.
